# Microsatellite Genotyping of *Plasmodium vivax* Isolates from Pregnant Women in Four Malaria Endemic Countries

**DOI:** 10.1371/journal.pone.0152447

**Published:** 2016-03-24

**Authors:** Michela Menegon, Azucena Bardají, Flor Martínez-Espinosa, Camila Bôtto-Menezes, Maria Ome-Kaius, Ivo Mueller, Inoni Betuela, Myriam Arévalo-Herrera, Swati Kochar, Sanjay K. Kochar, Puneet Jaju, Dhiraj Hans, Chetan Chitnis, Norma Padilla, María Eugenia Castellanos, Lucía Ortiz, Sergi Sanz, Mireia Piqueras, Meghna Desai, Alfredo Mayor, Hernando del Portillo, Clara Menéndez, Carlo Severini

**Affiliations:** 1 Department of Infectious, Parasitic and Immunomediated Diseases, Istituto Superiore di Sanità, Rome, Italy; 2 Barcelona Centre for International Health Research (CRESIB, Hospital Clínic-Universitat de Barcelona), Barcelona, Spain; 3 Gerência de Malária, Fundação de Medicina Tropical do Amazonas Dr. Heitor Vieira Dourado, Manaus, Brazil; 4 Universidade do Estado do Amazonas, Manaus, Amazonas, Brazil; 5 Papua New Guinea Institute of Medical Research, Goroka, Papua New Guinea; 6 Walter and Eliza Hall Institute, Parkville, Australia; 7 Centro Internacional de Vacunas / Faculty of Health, Universidad del Valle, Cali, Colombia; 8 Sardar Patel Medical College, Bikaner, India; 9 Malaria Group, International Centre for Genetic Engineering and Biotechnology, New Delhi, India; 10 Centro de Estudios en Salud, Universidad del Valle de Guatemala, Guatemala City, Guatemala; 11 Centers for Disease Control and Prevention, Center for Global Health, Division of Parasitic Diseases and Malaria, Atlanta, United States of America; 12 Institució Catalana de Recerca i Estudis Avançats, Barcelona, Spain; Centro de Pesquisa Rene Rachou/Fundação Oswaldo Cruz (Fiocruz-Minas), BRAZIL

## Abstract

*Plasmodium vivax* is the most widely distributed human parasite and the main cause of human malaria outside the African continent. However, the knowledge about the genetic variability of *P*. *vivax* is limited when compared to the information available for *P*. *falciparum*. We present the results of a study aimed at characterizing the genetic structure of *P*. *vivax* populations obtained from pregnant women from different malaria endemic settings. Between June 2008 and October 2011 nearly 2000 pregnant women were recruited during routine antenatal care at each site and followed up until delivery. A capillary blood sample from the study participants was collected for genotyping at different time points. Seven *P*. *vivax* microsatellite markers were used for genotypic characterization on a total of 229 *P*. *vivax* isolates obtained from Brazil, Colombia, India and Papua New Guinea. In each population, the number of alleles per locus, the expected heterozygosity and the levels of multilocus linkage disequilibrium were assessed. The extent of genetic differentiation among populations was also estimated. Six microsatellite loci on 137 *P*. *falciparum* isolates from three countries were screened for comparison. The mean value of expected heterozygosity per country ranged from 0.839 to 0.874 for *P*. *vivax* and from 0.578 to 0.758 for *P*. *falciparum*. *P*. *vivax* populations were more diverse than those of *P*. *falciparum*. In some of the studied countries, the diversity of *P*. *vivax* population was very high compared to the respective level of endemicity. The level of inter-population differentiation was moderate to high in all *P*. *vivax* and *P*. *falciparum* populations studied.

## Introduction

In endemic areas where *Plasmodium vivax* predominates, malaria in pregnancy is associated with detrimental effects on the health of the affected mothers and their infants [1–8]. Yet, there are still many gaps in the understanding of the mechanisms involved in the pathology of *P*. *vivax* infection in pregnancy. Genetic diversity in the *Plasmodium* populations is reported to be associated with the intensity of transmission. In *P*. *falciparum*, the genetic diversity is often, but not always, directly associated with transmission intensity [9–12]; in *P*. *vivax* the situation is more complicated and a number of studies reported high genetic diversity in parasite populations from low transmission settings [9,11,13–16]. As with *Plasmodium falciparum*, to understand the epidemiology, diversity, distribution and transmission dynamics of natural *P*. *vivax* populations in different epidemiological regions is crucial to develop specific control tools that target the distinctive biology of this neglected parasite. With malaria elimination back on the global agenda, mapping of global and local *P*. *vivax* population structure is essential prior to establishing goals for elimination and the roll out of interventions [17].

In recent years, reliable methods to genotype populations of *P*. *falciparum* and *P*. *vivax* have been developed. Genotyping methods based on the analysis of the polymorphic genes encoding antigens under immune selective pressure [18–20] might lead to a misunderstanding of the effective process of transmission [21]. Microsatellite (MS) markers, which are neutral or nearly neutral genetic markers, showed a high degree of allelic variation [22, 23] and are efficiently used for studies on genetic diversity and structure of both *P*. *falciparum* [24,25] and *P*. *vivax* populations [26]. Moreover, the use of MS markers can improve the capability to distinguish recrudescences/relapses from new infections in clinical trials [27].

The knowledge about the genetic variability of *P*. *vivax* is limited when compared to the information available for *P*. *falciparum*. Recent studies show that *P*. *vivax* parasites exhibit greater genetic diversity than *P*. *falciparum*, which suggests a greater functional variation, a distinct background of global colonization, and a more stable demographic history for *P*. *vivax* compared to *P*. *falciparum* [9, 11, 28–30]. Studies on malaria in pregnancy in Colombia and Thailand have recently shown that *P*. *vivax* parasites exhibited a high genetic diversity with similar level of expected heterozygosity in both pregnant and non-pregnant women as well as in symptomatic and asymptomatic infections [31, 32].

Here we present the results of a study that aimed to characterize the genetic structure of *P*. *vivax* populations obtained from pregnant women from four countries with different malaria endemic settings. Parasite isolates from three *P*. *falciparum* coendemic populations collected in pregnant women and *P*. *vivax* isolates from non-pregnant women collected in Brazil were also genotyped for comparison.

## Methods

### Ethics statement

Informed written consent was obtained from all subjects or their parents or guardians at the time of recruitment. The study protocol was reviewed and approved by the national and/or local ethics review boards in each of the sites, by the Institutional Review Board (IRB) of the Centers for Disease Control and Prevention (CDC), and by the Hospital Clínic of Barcelona Ethics Review Committee (CEIC). The study was conducted in accordance with the Good Clinical Practice Guidelines, the Declaration of Helsinki, and local rules and regulations of each partner country. The name of ethics review committees/boards at each study site and, when available, the respective approval numbers are listed as follows: Brasil-Comitê de Ética e Pesquisa da Fundação de Medicina Tropical do Amazonas, 0408/2008-FMT-AM, Comitê Nacional de Ética (CONEP), Brasilia, 0046.1.1144.000.08; Colombia -Comité Institucional de Revisión de Ética Humana. Facultad de Salud. Universidad del Valle. Cali, Colombia, 022–07; Guatemala-Comité de Ética, Universidad del Valle de Guatemala, Guatemala (no approval number assigned to the protocol); India-Ethics Committee Sandar Patel Medical College. Bikaner, Rajasthan, India (no approval number assigned to the protocol); Papua New Guinea (PNG)-PNG Institute of Medical Research Review Board, Madang, PNG, IRB 0815 MRAC 08.01.

### Study population and study areas

This study was conducted in the context of the PregVax project (FP7-HEALTH-Project Grant no. 201588), aimed at describing the burden and impact of *P*. *vivax* infection in pregnancy in five endemic areas (Brazil, Colombia, Guatemala, India and Papua New Guinea) of different malaria endemicity characteristics. The level of malaria transmission varied across study sites from hypoendemic in Guatemala, India and Colombia, mesoendemic in Brazil, to hyperendemic in Papua New Guinea. *P*. *vivax* was the predominant species in all sites except for Papua New Guinea where *P*. *vivax* co-existed with *P*. *falciparum* (50%) and other species (*P*. *malariae* and *P*. *ovale*, 5%) (S1 Table).

**Fig 1:**
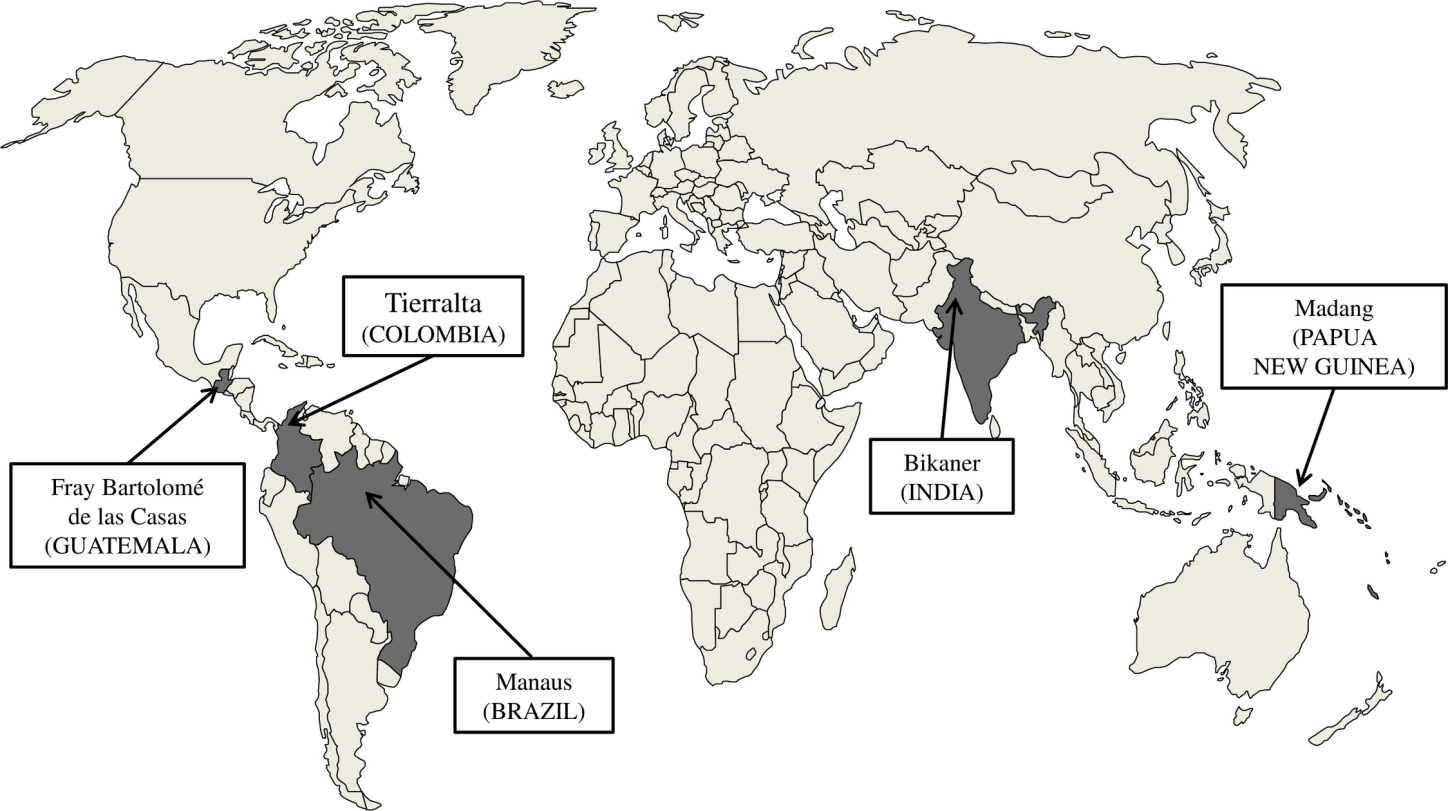
Map of the five endemic study areas that participated in the PregVax project.

Between June 2008 and October 2011 a cohort of nearly 2000 pregnant women were recruited at each site at recruitment visit, coinciding with an ANC visit, in two subsequent scheduled ANC visits one month apart and at delivery (the study sites for each country are shown in Fig 1).

A capillary blood sample was collected for *Plasmodium* infection determination by microscopy and by real time PCR method [33] from all study women. At delivery, placental blood, cord blood and newborn heel pricked blood samples were also collected (PregVax study group, unpublished data). The samples obtained from the Guatemala study area showed submicroscopic/very low parasitaemia. Because of this, very limited amount of DNA template was available to carry out further experiments, and therefore no data on MSs analyses were available to be included in the present study.

A total of 315 blood samples from *P*. *vivax* and *P*. *falciparum* infected pregnant women from Brazil, Colombia, India and Papua New Guinea were processed for genotyping.

Fifty-one samples from non-pregnant women were also collected in the community among women of reproductive age in Brazil. S2 Table summarizes the baseline characteristics of patients analyzed in our study.

### DNA extraction and microsatellite genotyping

Peripheral blood samples were spotted onto filter paper and analyzed at a centralized laboratory at the Istituto Superiore di Sanità in Rome, Italy, for *Plasmodium* determination by real time PCR method (PregVax multicenter study, unpublished data). A total of 229 *P*. *vivax* and 137 *P*. *falciparum* isolates from infected women were genotyped. DNA was extracted using PureLink Genomic DNA Kit (Invitrogen) following the manufacturer’s instructions. Seven *P*. *vivax* and six *P*. *falciparum* MS markers were used for the genotypic characterization of the field isolates (S3 Table). MS loci MS1, MS2, MS3, MS7, MS8, MS10 and MS20 were PCR-amplified from *P*. *vivax* isolates using labelled primers described by Karunaweera et al. 2007 [26].

The MSs Poly-α, TA60, Pfg377, PfPK2, TA87, TA109 were typed on *P*. *falciparum* positive samples as previously described [34], following the methodology developed by Anderson *et al*., 1999 [35]. Amplicon length variation of the PCR products was determined by CEQ 8000 Genetic analysis System (Beckman Coulter) using CEQ DNA Size Standard 400/600 as internal size standards and CEQ 8000 software for fragment analysis. The reported allele size was adjusted after comparison with reference alleles for each identified size in each locus. The exact sizes of the reference alleles were previously determined by direct sequencing.

### Microsatellite data and population genetic analyses

For each locus in each individual isolate, the predominant allele (where multiple alleles were detected) or the only allele (where only a single allele was scored) were counted for population genetic analyses. The predominant allele is defined as the highest peak in electropherogram traces.

Genetic variation for each MS locus in each population was assessed by calculating both the number of alleles per locus (A) and the expected heterozygosity (*H*_E_) from haploid data, as follows: *H*_E_ = [*n*/(*n* − 1)](1 − ∑*p*_*i*_^2^), where n is the number of isolates analyzed and p represents the frequency of each different allele at a locus. The potential range is from *H*_E_ = 0 (no allele diversity) to *H*_E_ = 1 (all sampled alleles are different). For isolates that were fully genotyped at all *P*. *vivax* and *P*. *falciparum* loci, analysis of multilocus linkage disequilibrium (LD) (non-random associations among loci) was assessed using the standardized index of association (*I*^*S*^_*A*_). Differences of the estimated mean *H*_E_ for each population and *I*^*S*^_*A*_ were calculated using the LIAN 3.5 software [36]. Additionally, allelic richness (Rs), which is a measure of the number of alleles independent of sample size, was estimated per each country using FSTAT software v2.9.3.2 [37]. Genetic differentiation between populations (*F*_ST_) was estimated using Wright's F statistics, and calculations were computed by Arlequin 3.5 [38]. Differences between the mean *H*_E_ of any two groups for each country was assessed using the Mann-Whitney U test. Differences were considered significant if the calculated p value was ≤0.05 (two-tailed test).

## Results

Complete results for all *P*. *vivax* and *P*. *falciparum* MS analyzed loci are shown as supporting information in S1 Dataset. Most part of missing MSs data was linked to the high percentage of the *P*. *vivax* and *P*. *falciparum* submicroscopic infections detected in the analyzed sample subset.

### Genetic diversity and differentiation among *Plasmodium vivax* populations

A total of 88 *P*. *vivax* isolates from Brazil, 47 from Colombia, 21 from India and 73 from Papua New Guinea were typed for MS loci 1 to 7 (S1 Dataset). All the isolates from Colombia, India, Papua New Guinea, and 37 isolates from Brazil were collected from pregnant women at the time of ANC visits or at delivery. Fifty-one isolates from were obtained from non-pregnant women.

**Fig 2:**
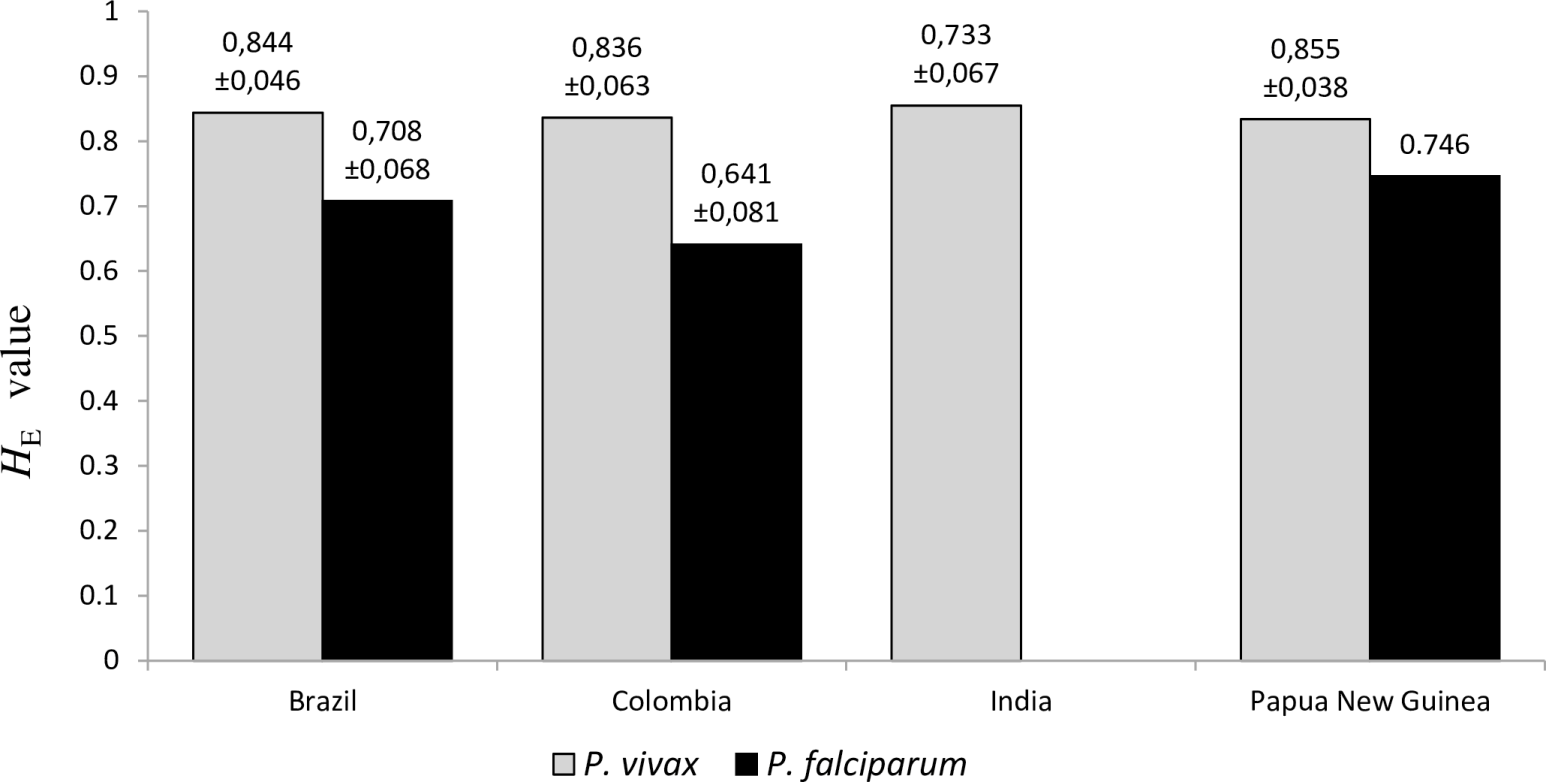
Genetic diversity (*H*_E_) of analyzed *P*. *vivax* and *P*. *falciparum* populations.

Full allelic data for all 7 MS loci were obtained for 133 of the total 229 analyzed *P*. *vivax* isolates (Table 1), leading to the identification of 129 different haplotypes, with none of the haplotypes shared between the parasite populations of the four endemic settings. The total number of distinct alleles observed per locus ranged from a total of 35 for MS8 to 10 for MS1 and MS3, with an overall mean of 23.1 alleles/ locus and a mean value of 11 alleles/country. Strong genetic diversity and high values of allelic richness were observed across all analyzed study sites. In particular, the isolates from India revealed the greatest level of genetic diversity (mean *H*_E_ = 0.855) whereas the lowest one was observed in Papua New Guinea (mean *H*_E_ = 0.834) (Fig 2). The isolates from Brazil showed the highest mean value of allelic richness (mean Rs = 12.02). In particular, Brazilian isolates from the non-pregnant group (N = 37; *H*_E_ = 0.841) displayed a mean Rs value of 8.94, whereas isolates from pregnant group (N = 11, *H*_E_ = 0.805) showed an Rs value of 6.28 (S4 Table).

**Table 1:** Allelic richness (Rs) and LD (*I^S^_A_*) of analyzed *Plasmodium vivax* populations.

Country	No. of isolates*	No. of haplotypes	Mean no. of alleles	Rs^§^	I_A_^S^ (p#)	
**Brazil**	**88/48 (50%)**	**46**	**14.1**	**12.02**	**0.0648 (< 0.0001)**	
**Colombia**	**47/22 (46.8%)**	**22**	**10.1**	**10.13**	**0.0272 (0.0629)**	
**India**	**21/21 (100%)**	**21**	**9.8**	**9.85**	**0.0739 (0.0005)**	
**PNG**	**73/42 (57.5%)**	**40**	**10.7**	**9.59**	**0.0405 (<0.0001)**	

* Number of isolates genotyped for at least 1 MS / number of isolates fully genotyped at all 7 MS loci (%)

^§^ Rs mean is based on minimum sample size of 21 individuals

^#^ LD (*I*^*S*^_*A*_) is considered significant when p value is ≤0.05

We compared the mean *H*_E_ values from Brazilian pregnant and non-pregnant groups but no significant difference was found (Mann-Whitney U test, p = 0.898); similarly, no significant difference (p = 0.126) was found comparing Rs values from the same subgroups.

The analyzed *P*. *vivax* populations showed a significant LD (*I*^*S*^_*A*_ > 0; p < 0.001) at multiple loci in each population except for the case of Colombia (p = 0.062). A moderate degree of LD was observed in Brazil, India and Papua New Guinea (Table 1).

Levels of genetic differentiation between each possible pair of the four parasite populations are indicated by *F*_ST_ values in Table 2. All values were significantly different from 0 (p < 0.05). We observed the highest differentiation between Colombia and Papua New Guinea (*F*_ST_ = 0.102), whereas India and Brazil displayed the lowest value (*F*_ST_ = 0.045) of the analyzed *vivax* populations.

**Table 2:** Genetic distance (*F*_ST_) between *P*. *vivax* populations across study sites.

	*F*_ST*_		
Country	Brazil	Colombia	India
**Brazil**	---		
**Colombia**	0.06853	---	
**India**	0.04540	0.07859	---
**PNG**	0.09109	0.10264	0.06485
** **			

*F*_ST_*: all values resulting from the pairwise comparison of each country are significant (p < 0.05)

### Genetic diversity and differentiation among *Plasmodium falciparum* populations

*P*. *falciparum* populations were also investigated in three of the study sites for comparison at six MS loci. The genotyping was performed in 137 *P*. *falciparum* positive blood samples obtained from pregnant women in Brazil (n = 15), Colombia (n = 50) and Papua New Guinea (n = 72) (S1 Dataset) and, among these, a total of 66 isolates were fully genotyped as shown in Table 3. None of the total 55 identified haplotypes were shared among the three *P*. *falciparum* parasite populations. Estimates of *H*_E_ and Rs per country were calculated (Table 3, Fig 2). The highest estimates of genetic diversity were observed in samples obtained from pregnant women in Papua New Guinea and the lowest in Colombia. Comparing the mean number of alleles and Rs values obtained in Brazil with whose obtained in Colombia, we found similar values, even if the *P*. *falciparum* populations from Brazil revealed a slightly higher of genetic diversity. Among *P*. *falciparum* analyzed MS loci, locus TA60 showed the same two alleles in the total 50 isolates from Colombia and in the total 15 isolates from Brazil. Additionally, locus TA60 displayed a monomorphic allele in all 27 fully typed Colombian isolates. No LD, measured by *I*^*S*^_*A*_ index, was observed in Papua New Guinea (*I*^*S*^_*A*_ > 0.0232; p = 0.06) while a significantly high LD value was found both in Brazil and Colombia (Table 3), however, the small number of tested isolates from Brazil (n = 8) may produce a biased *I*^*S*^_*A*_ value.

**Table 3:** Allelic richness (Rs) and LD (*I^S^_A_*) of analyzed *Plasmodium falciparum* populations.

Country	No. of isolates*	No. of haplotypes	Mean no. of alleles	Rs^§^	*I*^*S*^_*A*_ (p^#^)
**Brazil**	15/8 (53.3%)	8	3.8	3.8	0.1718 (0.0094)
**Colombia**	50/27 (54%)	20	3.5	3.1	0.0354 (0.0292)
**PNG**	72/31 (43%)	27	6.3	5	0.0232 (0.0607)

* Number of isolates genotyped for at least 1 MS / number of isolates fully genotyped at all 6 MS loci (%)

§ Rs mean is based on minimum samples size of 8 individuals

^**#**^LD (*I*^*S*^_*A*_) is considered significant when p value is ≤0,05

The greatest value of inter-population differentiation (*F*_ST_ = 0.252, p< 0.05) was found between Brazil and Colombia. In Papua New Guinea isolates displayed a moderate level of significant differentiation, about 14.7% and 12.9% of the variation found in the Brazil and Colombia populations, respectively (Table 4).

**Table 4:** Genetic distance (*F*_*ST*_) between *Plasmodium falciparum* populations from three analyzed countries.

Country	F_ST_*	
	Brazil	Colombia
**Brazil**	---	
**Colombia**	0.25216	---
**PNG**	0.14781	0.12901

*F*_ST_*: all values resulting from the pairwise comparison of each country are significant (p < 0.05)

### Comparison of haplotypes from different time points and compartments

Complete genotyping data were also obtained for isolates collected from successive samples during the follow up of 9 pregnant women with *P*. *vivax* infection. In particular, in 4 infected women from Colombia and in 2 from Papua New Guinea, a distinct parasite haplotype was detected in each of the subsequent episodes that occurred between 4 to 9 weeks after the first episode. In 2 infected women from Colombia (by comparing 6 MS loci successfully genotyped) and in 1 from Brazil the same haplotype was identified in subsequent episodes occurring at 3, 11 and 17 weeks.

In addition, parasite haplotypes from maternal peripheral blood at delivery and the placenta were compared in 4 women (2 from Colombia and 2 from Papua New Guinea) infected with *P*. *falciparum* infection. In all cases, parasites from maternal and placental blood had an identical MS profile.

### Genetic diversity according to *Plasmodium* species, age and parity of the host

Only in Colombia we detected a significantly higher mean *H*_E_ value among pregnant women infected with *P*. *vivax* compared to those with *P*. *falciparum* infection (Mann-Whitney U test, p = 0.027). Additionally, the genetic diversity of *Plasmodium* isolates according to age, parity, parasitaemia (microscopic or submicroscopic) and presence of fever (≥37.5°C) among the infected women were compared (sample size for each compartment is shown in S5 Table). In Brazil, a significantly higher genetic diversity was observed in *P*. *vivax* parasites infecting multigravidae (p = 0.003), those ≥ 25 years old (p = 0.009) and those who were afebrile (p = 0.015), compared to primigravidae, those aged 11–20 years old and febrile women, respectively. In contrast, *P*. *falciparum* parasites infecting ≥ 25 years old (p = 0.003) and those who were afebrile (p = 0.032), showed a significantly lower genetic diversity, while significant difference was not observed in the genetic diversity level for other analyzed subgroups and countries. In Colombia, the situation was exactly the opposite of what was observed in Brazil, but the differences in *H*_*E*_ values were not statistically significant. In Papua New Guinea, the genetic diversity was very similar in each age class for both *vivax* and *falciparum*, and the slight differences observed were not significant in Mann-Whitney U test.

## Discussion

This study was aimed at characterizing the genetic diversity of *P*. *vivax* populations in blood samples obtained from pregnant women across different endemic areas by MS analysis. Overall, a wide range of short tandem repeat variants and high level of diversity in *P*. *vivax* and also *P*. *falciparum* were observed. In particular, for each of the countries included in the study, the diversity of *P*. *vivax* populations was slightly higher than for *P*. *falciparum* populations, though a significant difference between *H*_*E*_ values for the two species was only found in Colombian isolates.

By evaluating these data according to the level of malaria endemicity, we know that the investigated areas in Colombia and India show a low level of endemicity; Manaus, in Brazil, is considered mesoendemic whereas Madang (Papua New Guinea) is the only area with a high level of endemicity. *P*. *falciparum* populations show a direct association between the genetic diversity and transmission intensity. However, the situation for *P*. *vivax* is more complicated with observations showing high genetic diversity in parasite populations from low transmission settings [9–16]. Findings of the present study relevant to the level of genetic diversity per country/per species (Fig 2), are consistent with what has been reported so far in the literature. Indeed, *falciparum* population from Papua New Guinea (hyperendemic country) showed the highest value of *H*_*E*_ in comparison with the other populations studied, while high values of *H*_*E*_ were found in *vivax* populations from India and Colombia (hypoendemic) and Brazil (mesoendemic), confirming that this parasite is able to maintain a high degree of genetic variability irrespective of the transmission situation of its endemic area. Actually, the lowest *H*_*E*_ value among the *vivax* population included in this study was found in the parasite population from Papua New Guinea, which happens to be the country with a high malaria transmission intensity.

Based on previously defined range of genetic differentiation [39–40], almost all *P*. *vivax* populations examined showed moderate level of differentiation ranging from about 6.4% to 10.2% (*F*_ST_ pairwise analysis). On the contrary, *falciparum* populations showed to be highly differentiated from each other in the pairwise comparison between countries (*F*_ST_, from 12.9% to 25.2%); parasites from the two geographically closer Southern American countries, Brazil and Colombia, appeared to be the most divergent according to the *F*_ST_ analysis (25.2%). Our result is consistent with the independent origins of these two parasite populations, as shown by Yalcindag et al (2012) [41]. Indeed, the authors speculated that South American *P*. *falciparum* populations are subdivided into two main genetic clusters: northern (including Colombia) and southern (Brazil, Bolivia and French Guiana), due to two different introduction events during European colonization.

Moreover, the study allowed comparison of genetic variability according to parity, age, level of parasitaemia, or presence of fever both in *P*. *vivax* and *P*. *falciparum* populations from Brazil, Colombia and Papua New Guinea. Interestingly, for the *P*. *vivax* population from Brazil, we have noticed that older age was associated with a significant increase in *H*_E_ values, whereas for the Brazilian *P*. *falciparum* population an inverse relationship to age was observed. A possible explanation for this opposite trend in the two *Plasmodium* species might be that the different level of endemicity for *P*. *vivax* and *P*. *falciparum* populations could result in differences in reaching acquired immunity (in most of *P*. *vivax* low endemic areas this is rarely achieved even during the course of a lifetime [42]) and this, in turn, could affect the number of plasmodial clones circulating in a given area and hence the level of parasite genetic diversity. A decrease of genetic diversity with older age has been already observed in *P*. *falciparum* populations and reported in previous studies carried out in Sudan and Central African Republic [43, 44]. In Colombia, the situation was exactly the opposite of what was observed in Brazil, but the differences in *H*_*E*_ values were not statistically significant. In Papua New Guinea, the genetic diversity was very similar in each age class for both *vivax* and *falciparum*, and the slight differences observed were not significant.

Malaria infection in pregnancy is associated with high risk of both maternal and perinatal morbidity and mortality. It is recognized that pregnant women have a reduced immune response and therefore clear malaria parasites with difficulty and are more exposed to the severe form of the diseases [45]. In our study, only in Brazil it was possible to investigate whether *Plasmodium* isolates detected in pregnant infected women could show different genotypic characteristics compared to non-pregnant women. High *H*_*E*_ values were observed in the Brazilian *P*. *vivax* populations both from pregnant (0.805) and non-pregnant (0.841) infected women and the differences between the two *H*_E_ values were not statistically significant. Similar findings have been recently described in Colombia [31]. The observed LD values were quite different however the *vivax* populations from pregnant women included a small number of samples (n = 11), which may have affected the evaluation of LD in this subpopulation.

By genotyping nine patients with subsequent *P*. *vivax* episodes, we observed that the genotype of the parasites of the admission episode can differ or can be identical to the genotype of the subsequent episode and that these results are in line to what was reported in previous studies [46–49]. Hence, in the attempt to distinguish between relapses, recrudescences and new infections, the level of transmission in the area, the treatment regimen received and the time intervals between the episodes have to be taken into account together with the results of genetic characterization of parasite isolates [47]. In our study we observed four subsequent episodes in four Colombian patients that can be attributable to relapses. In these patients, the isolate identified in the admission episode had a different MS haplotype compared to that one identified in the corresponding subsequent episode. Taking into account the level of malaria endemicity in Colombia (hypoendemicity) as well as the time interval between the admission episode and the subsequent episode, we can assume that the four patients had relapses due the activation of latent hypnozoites, showing a different haplotype rather than a new infection.

In the frame of Pregvax project, we have also identified and genotyped two cases of congenital malaria, a case in a woman from Guatemala infected with *P*. *vivax* (see reference [50]) and a case in a woman from Papua New Guinea, infected with *P*. *falciparum*. In both cases, identical parasite haplotypes in maternal peripheral blood at delivery and in the placental, cord and newborn peripheral blood were found. In our knowledge, these are the only two cases where mother-to-child malaria transmission has been confirmed by means of MS analysis.

The findings of this study confirm the usefulness of MS analysis as a genetic tool for investigating *P*. *vivax* and *P*. *falciparum* population structures. As already reported in previous similar studies [9, 28, 29, 51], *P*. *vivax* populations were more diverse than those of *P*. *falciparum* and in some of the studied countries, the diversity of *P*. *vivax* populations was striking compared to the respective level of endemicity. Molecular surveillance of the *Plasmodium* genetic variability is of great importance in the context of malaria containment efforts, since a correct and extensive appraisal of the genetic makeup of plasmodial populations is crucial in the identification of the most adequate control strategies, particularly in countries that are in the pathway towards malaria elimination. In the last few years, many studies have included the investigation of the genetic variability of *P*. *vivax* isolates through the analysis of the MS diversity, however, those including these analysis in pregnancy are scarce [31,32]. For this reason the present study, which included samples mainly from pregnant women, from endemic areas of four different countries, could contribute to increasing the knowledge on the genetic diversity of parasite populations of this important but still neglected human malaria parasite.

## Supporting Information

S1 Dataset Complete results for all *P*. *vivax* and *P*. *falciparum* MS analyzed loci. (XLSX)

S1 Table Characteristics of the study sites. (TIF)

S2 Table Baseline characteristics of patients analyzed in our study. (TIF)

S3 Table Molecular markes and PCR conditions used in the present study. (TIF)

S4 Table Genetic diversity, allelic richness, linkage disequilibrium and genetic distance of Brazilian *P*. *vivax* subpopulations. (TIF)

S5 Table Number of infected pregnant women with available data for each compartment. (TIF)

## References

[1] Rodriguez-Morales AJ, Sanchez E, Vargas M, Piccolo C, Colina R, Arria M, et al Pregnancy outcomes associated with *Plasmodium vivax* malaria in northeastern Venezuela. Am J Trop Med Hyg. 2006; 74:755–757. 16687675

[2] Nosten F, McGready R, Simpson JA, Thwai KL, Balkan S, Cho T, et al Effects of *Plasmodium vivax* malaria in pregnancy. Lancet.1999; 354:546–549. 10470698 10.1016/s0140-6736(98)09247-2

[3] Martinez-Espinosa FE, Daniel-Ribeiro CT, Alecrim WD. Malaria during pregnancy in a reference centre from the Brazilian Amazon: unexpected increase in the frequency of *Plasmodium falciparum* infections. Mem Inst Oswaldo Cruz 2004; 99:19–21. 15057341 10.1590/s0074-02762004000100003

[4] Singh N, Shukla MM, Sharma VP. Epidemiology of malaria in pregnancy in central India. Bull World Health Organ.1999; 77:567–572. 10444880 PMC2557706

[5] Sholapurkar SL, Gupta AN, Mahajan RC. Clinical course of malaria in pregnancy-a prospective controlled study from India. Trans R Soc Trop Med Hyg. 1988; 82:376–379. 3068848 10.1016/0035-9203(88)90124-1

[6] Carvalho BO, Matsuda JS, Luz SL, Martinez-Espinosa FE, Leite JA, Franzin F et al Gestational malaria associated to *Plasmodium vivax* and *Plasmodium falciparum* placental mixed-infection followed by fetal loss: a case report from an unstable transmission area in Brazil. Malar J. 2011:10:178 10.1186/1475-2875-10-178 21708032 PMC3141593

[7] Singh N, Singh MP, Wylie BJ, Hussain M, Kojo YA, Shekhar C et al Malaria prevalence among pregnant women in two districts with differing endemicity in Chhattisgarh, India. Malar J. 2012; 11:274 10.1186/1475-2875-11-274 22882903 PMC3489539

[8] Singh N, Saxena A, Shrivastava R. Placental *Plasmodium vivax* infection and congenital malaria in central India. Ann Trop Med Parasitol. 2003; 97:875–878. 14754501 10.1179/000349803225002688

[9] Orjuela-Sánchez P, Sá JM, Brandi MC, Rodrigues PT, Bastos MS, Amaratunga C et al Higher microsatellite diversity in *Plasmodium vivax* than in sympatric *Plasmodium falciparum* populations in Pursat, Western Cambodia. Exp Parasitol. 2013; 134:318–326. 10.1016/j.exppara.2013.03.029 23562882 PMC3691688

[10] Nkhoma SC, Nair S, Al-Saai S, Ashley E, McGready R, Phyo AP, et al Population genetic correlates of declining transmission in a human pathogen. Mol Ecol.2013; 22:273–285. 10.1111/mec.12099 23121253 PMC3537863

[11] Noviyanti R, Coutrier F, Utami RA, Trimarsanto H, Tirta YK, Trianty L, et al Contrasting Transmission Dynamics of Co-endemic *Plasmodium vivax* and *P*. *falciparum*: Implications for Malaria Control and Elimination. PLoS Negl Trop Dis. 2015; 9:e0003739 10.1371/journal.pntd.0003739 25951184 PMC4423885

[12] Escalante AA, Ferreira MU, Vinetz JM, Volkman SK, Cui L, Gamboa D, et al Malaria Molecular Epidemiology: Lessons from the International Centers of Excellence for Malaria Research Network. Am J Trop Med Hyg. 2015; 93:79–86. 10.4269/ajtmh.15-0005 26259945 PMC4574277

[13] Abdullah NR, Barber BE, William T, Norahmad NA, Satsu UR, Muniandy PK et al *Plasmodium vivax* population structure and transmission dynamics in Sabah Malaysia. PLoS One.2013; e82553 10.1371/journal.pone.0082553 24358203 PMC3866266

[14] Iwagami M, Fukumoto M, Hwang SY, Kim SH, Kho WG, Kano S. Population structure and transmission dynamics of *Plasmodium vivax* in the Republic of Korea based on microsatellite DNA analysis. PLoS Negl Trop Dis. 2012; 6:e1592 10.1371/journal.pntd.0001592 22509416 PMC3317904

[15] Rezende AM, Tarazona-Santos E, Fontes CJ, Souza JM, Couto AD, Carvalho LH et al Microsatellite loci: determining the genetic variability of *Plasmodium vivax*. Trop Med Int Health. 2010;15:718–26. 10.1111/j.1365-3156.2010.02535.x 20406424

[16] Koepfli C, Rodrigues PT, Antao T, Orjuela-Sánchez P, Van den Eede P, Gamboa D et al *Plasmodium vivax* Diversity and Population Structure across Four Continents. PLoS Negl Trop Dis. 2015; 9:e0003872 10.1371/journal.pntd.0003872 26125189 PMC4488360

[17] Arnott A, Barry AE, Reeder JC. Understanding the population genetics of *Plasmodium vivax* is essential for malaria control and elimination. Malar J. 2012; 11:14 10.1186/1475-2875-11-14 22233585 PMC3298510

[18] Joshi H. Markers for population genetic analysis of human plasmodia species, *P*. *falciparum* and *P*. *vivax*. J Vector Borne Dis. 2003; 40:78–83. 15119076

[19] Hughes MK, Hughes AL. Natural selection on *Plasmodium s*urface proteins. Mol Biochem Parasitol. 1995; 71:99–113. 7630387 10.1016/0166-6851(95)00037-2

[20] Escalante AA, Lal AA, Ayala FJ. Genetic polymorphism and natural selection in the malaria parasite *Plasmodium falciparum*. Genetics. 1998; 149:189–202. 9584096 10.1093/genetics/149.1.189 PMC1460124

[21] Cattamanchi A, Kyabayinze D, Hubbard A, Rosenthal PJ, Dorsey G. Distinguishing recrudescence from reinfection in a longitudinal antimalarial drug efficacy study: comparison of results based on genotyping of msp-1, msp-2, and glurp. Am J Trop Med Hyg. 2003; 68:133–139. 12641400

[22] Su Xz, Wellems TE. Toward a high-resolution *Plasmodium falciparum* linkage map: polymorphic markers from hundreds of simple sequence repeats. Genomics. 1996; 33:430–444. 8661002 10.1006/geno.1996.0218

[23] Brito CF, Ferreira MU. Molecular markers and genetic diversity of *Plasmodium vivax*. Mem Inst Oswaldo Cruz. 2011; 106 Suppl 1:12–26. 21881753 10.1590/s0074-02762011000900003

[24] Anderson TJ, Haubold B, Williams JT, Estrada-Franco JG, Richardson L, Mollinedo R, et al Microsatellite markers reveal a spectrum of population structures in the malaria parasite *Plasmodium falciparum*. Mol Biol Evol. 2000; 17:1467–1482. 11018154 10.1093/oxfordjournals.molbev.a026247

[25] Su X, Hayton K, Wellems TE. Genetic linkage and association analyses for trait mapping in *Plasmodium falciparum*. Nat Rev Genet. 2007; 8:497–506. 17572690 10.1038/nrg2126

[26] Karunaweera ND, Ferreira MU, Hartl D, Wirth DF. Fourtheen polymorphic microsatellite DNA markers for the human malaria parasite *Plasmodium vivax*. Mol Ecol Notes.2007; 7:172–175

[27] Koepfli C, Mueller I, Marfurt J, Goroti M, Sie A, Oa O, et al Evaluation of *Plasmodium vivax* genotyping markers for molecular monitoring in clinical trials. J Infect Dis. 2009; 199:1074–1080. 10.1086/597303 19275476

[28] Neafsey DE, Galinsky K, Jiang RH, Young L, Sykes SM, Saif S et al The malaria parasite *Plasmodium vivax* exhibits greater genetic diversity than *Plasmodium falciparum*. Nat Genet. 2012; 44:1046–1050. 10.1038/ng.2373 22863733 PMC3432710

[29] Gray KA, Dowd S, Bain L, Bobogare A, Wini L, Shanks GD, et al Population genetics of Plasmodium falciparum and Plasmodium vivax and asymptomatic malaria in Temotu Province, Solomon Islands. Malar J. 2013; 12:429 10.1186/1475-2875-12-429 24261646 PMC4222835

[30] Jennison C, Arnott A, Tessier N, Tavul L, Koepfli C, Felger I, et al *Plasmodium vivax* populations are more genetically diverse and less structured than sympatric *Plasmodium falciparum* populations. PLoS Negl Trop Dis. 2015; 9(4):e0003634 10.1371/journal.pntd.0003634 25874894 PMC4398418

[31] Arango EM, Samuel R, Agudelo OM, Carmona-Fonseca J, Maestre A, Yanow SK. Genotype comparison of *Plasmodium vivax* and *Plasmodium falciparum* clones from pregnant and non-pregnant populations in North-west Colombia. Malar J. 2012; 11:392 10.1186/1475-2875-11-392 23181896 PMC3519599

[32] Thanapongpichat S, McGready R, Luxemburger C, Day NP, White NJ, Nosten F et al Microsatellite genotyping of *Plasmodium vivax* infections and their relapses in pregnant and non-pregnant patients on the Thai-Myanmar border. Malar J. 2013; 12:275 10.1186/1475-2875-12-275 23915022 PMC3750759

[33] Veron V, Simon S, Carme B. Multiplex real-time PCR detection of *P*. *falciparum*, *P*. *vivax* and *P*. *malariae* in human blood samples. Exp Parasitol. 2009; 121: 346–351. 10.1016/j.exppara.2008.12.012 19124021

[34] Pumpaibool T, Arnathau C, Durand P, Kanchanakhan N, Siripoon N, Suegorn A et al Genetic diversity and population structure of *Plasmodium falciparum* in Thailand, a low transmission country. Malar J. 2009; 8:155 10.1186/1475-2875-8-155 19602241 PMC2722663

[35] Anderson TJ, Su XZ, Bockarie M, Lagog M, Day KP. Twelve microsatellite markers for characterization of *Plasmodium falciparum* from finger-prick blood samples. Parasitology. 1999; 119:113–125. 10466118 10.1017/s0031182099004552

[36] Haubold B, Hudson RR. LIAN 3.0: detecting isequilibrium in multilocus data. Linkage Analysis Bioinformatics. 2000; 16:847–848. 11108709 10.1093/bioinformatics/16.9.847

[37] Goudet J. FSTAT (Version 1.2): a computer program to calculate F-statistics. J. Heredity. 1995; 86:485–6.

[38] Excoffier L, Laval G, Schneider S. Arlequin (version 3.0): an integrated software package for population genetics data analysis. Evol Bioinform Online. 2005; 1:47–50. PMC2658868 19325852

[39] Wright S. Evolution and the Genetics of Population Vol. 4, Variability Within and Among Natural Populations. The University of Chicago Press; 1978.

[40] Hartl DL, Clark AG. Principles of Population Genetics 3nd edn. Sinauer Associates, Inc, Sunderland, MA; 1997

[41] Yalcindag E, Elguero E, Arnathau C, Durand P, Akiana J, Anderson TJ et al Multiple independent introductions of *Plasmodium falciparum* in South America. Proc Natl Acad Sci U S A. 2012; 109:511–516. 10.1073/pnas.1119058109 22203975 PMC3258587

[42] Mendis K, Sina BJ, Marchesini P, Carter R. The neglected burden of *Plasmodium vivax* malaria. Am J Trop Med Hyg. 2001; 64:97–106. 11425182 10.4269/ajtmh.2001.64.97

[43] Hamid MM, Mohammed SB, El Hassan IM. Genetic diversity of *Plasmodium falciparum* field isolates in Central Sudan inferred by pcr genotyping of merozoite surface protein 1 and 2. n am j med sci. 2013; 5:95–101. 10.4103/1947-2714.107524 23641369 PMC3624726

[44] Dolmazon V, Matsika-Claquin MD, Manirakiza A, Yapou F, Nambot M, Menard D. Genetic diversity and genotype multiplicity of *Plasmodium falciparum* infections in symptomatic individuals living in Bangui (CAR). Acta Trop. 2008; 107:37–42. 10.1016/j.actatropica.2008.04.012 18501320

[45] Takem EM, D’Alessandro U. Malaria in pregnancy. Mediterr J Hematol Infect Di. 2013; 5:e2013010 10.4084/MJHID.2013.010 PMC3552837 23350023

[46] Chen N, Auliff A, Rieckmann K, Gatton M, Cheng Q. Relapses of *Plasmodium vivax* infection result from clonal hypnozoites activated at predetermined intervals. J Infect Dis. 2007; 195: 934–41. 17330782 10.1086/512242

[47] Imwong M, Snounou G, Pukrittayakamee S, Tanomsing N, Kim JR, Nandy A, et al Relapses of *Plasmodium vivax* infection usually result from activation of heterologous hypnozoites. J Infect Dis. 2007; 195:927–933. 17330781 10.1086/512241

[48] Imwong M, Boel ME, Pagornrat W, Pimanpanarak M, McGready R, Day NP, et al The first *Plasmodium vivax* relapses of life are usually genetically homologous. J Infect Dis. 2012; 205:680–3. 10.1093/infdis/jir806 22194628 PMC3266132

[49] de Araujo FC, de Rezende AM, Fontes CJ, Carvalho LH, Alves de Brito CF. Multiple-clone activation of hypnozoites is the leading cause of relapse in *Plasmodium vivax* infection. PLoS One.2012; 7(11):e49871 10.1371/journal.pone.0049871 23185469 PMC3503861

[50] Castellanos ME, Bardaji A, Menegon M, Mayor A, Desai M, Severini C, et al *Plasmodium vivax* congenital malaria in an area of very low endemicity in Guatemala: implications for clinical and epidemiological surveillance in a malaria elimination context. Malar J. 2012; 11:411 10.1186/1475-2875-11-411 23217209 PMC3541160

[51] Gunawardena S, Ferreira MU, Kapilananda GM, Wirth DF, Karunaweera ND. The Sri Lankan paradox: high genetic diversity in *Plasmodium vivax* populations despite decreasing levels of malaria transmission. Parasitology. 2014;141:880–90 10.1017/S0031182013002278 24533989 PMC7485621

