# Supplementary figures and images for: Microsatellite Genotyping of Plasmodium vivax Isolates from Pregnant Women in Four Malaria Endemic Countries
Source: PLoS One. 2016 Mar 24;11(3):e0152447. doi: 10.1371/journal.pone.0152447 (PMC4807005; doi:10.1371/journal.pone.0152447)

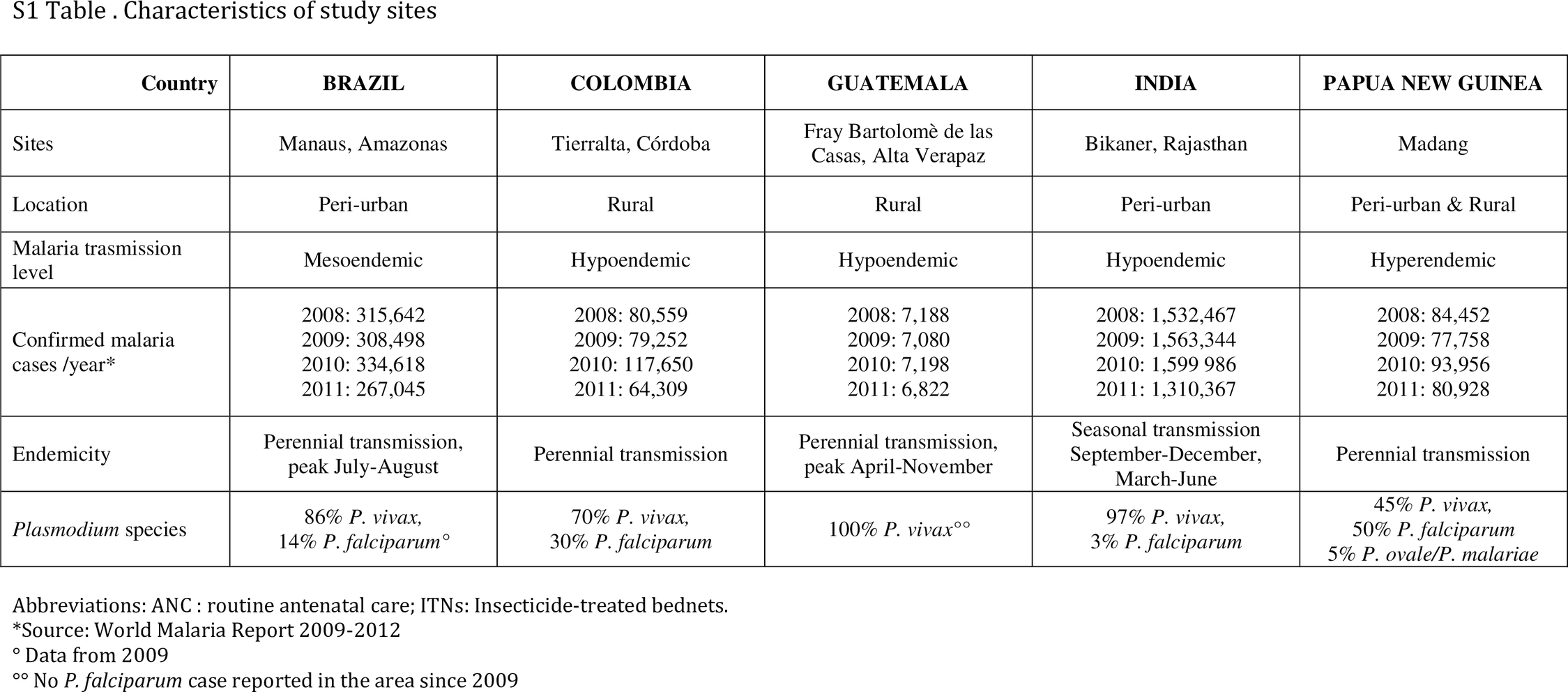

Supplement: S1 Table — (TIF) [file pone.0152447.s002.tif]

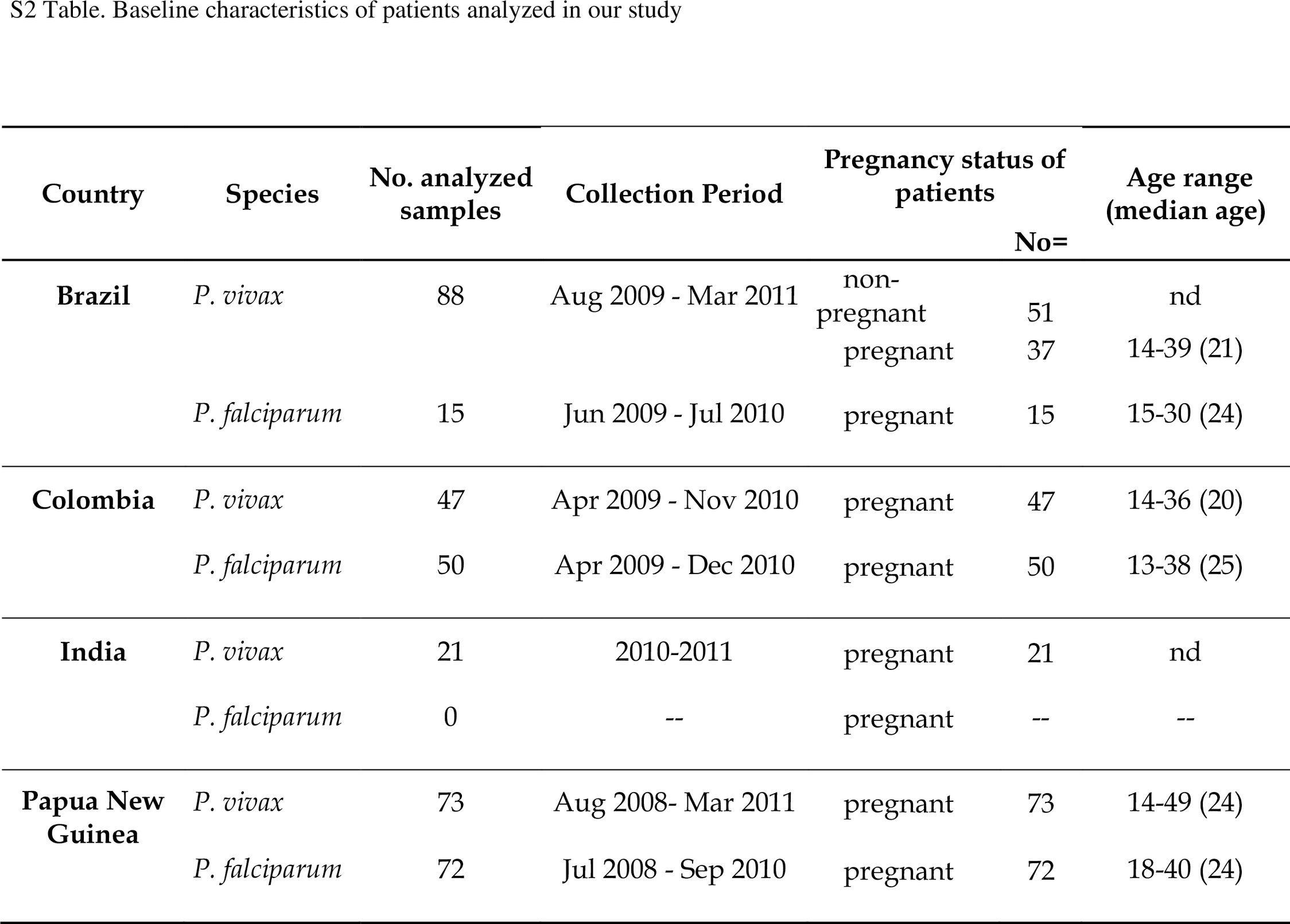

Supplement: S2 Table — (TIF) [file pone.0152447.s003.tif]

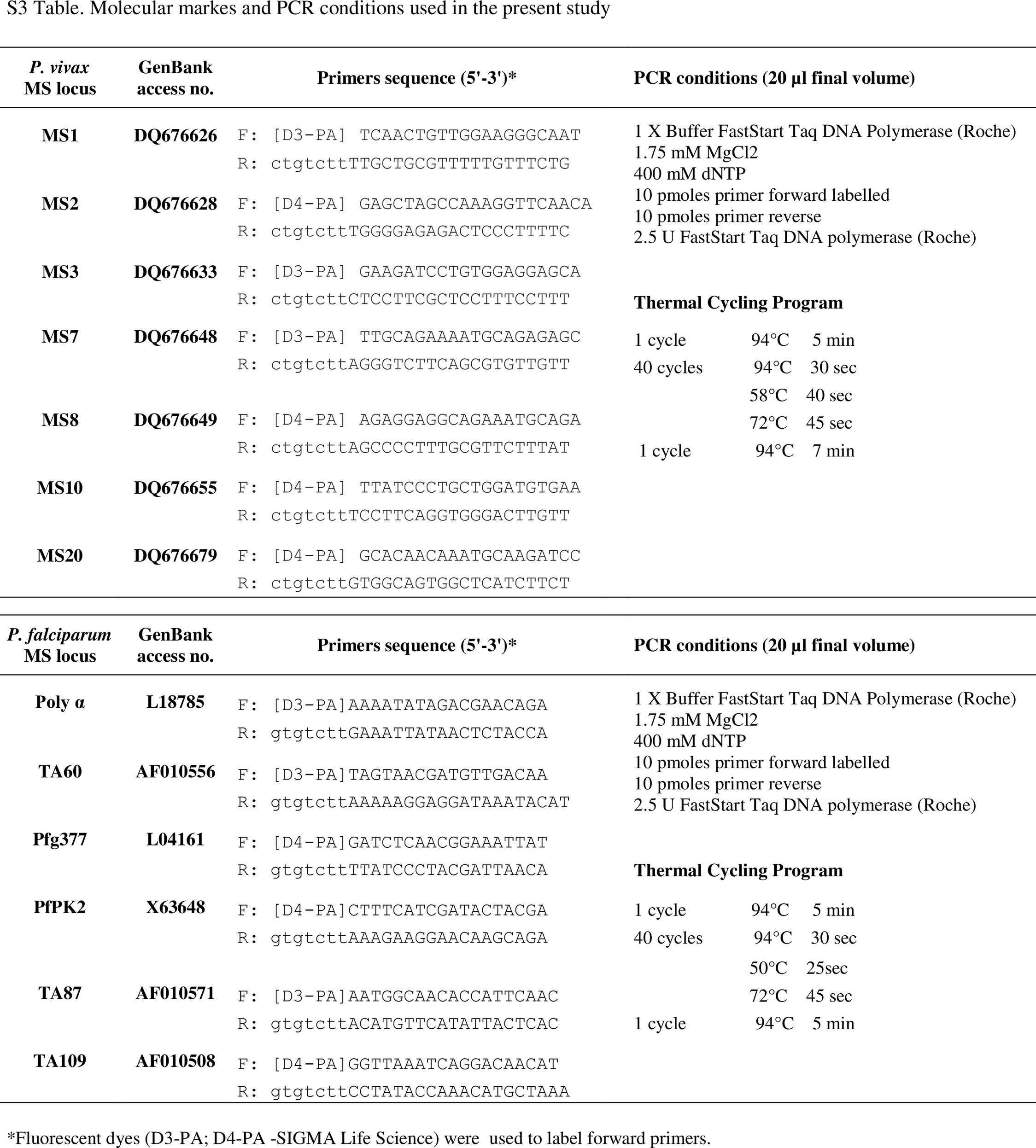

Supplement: S3 Table — (TIF) [file pone.0152447.s004.tif]

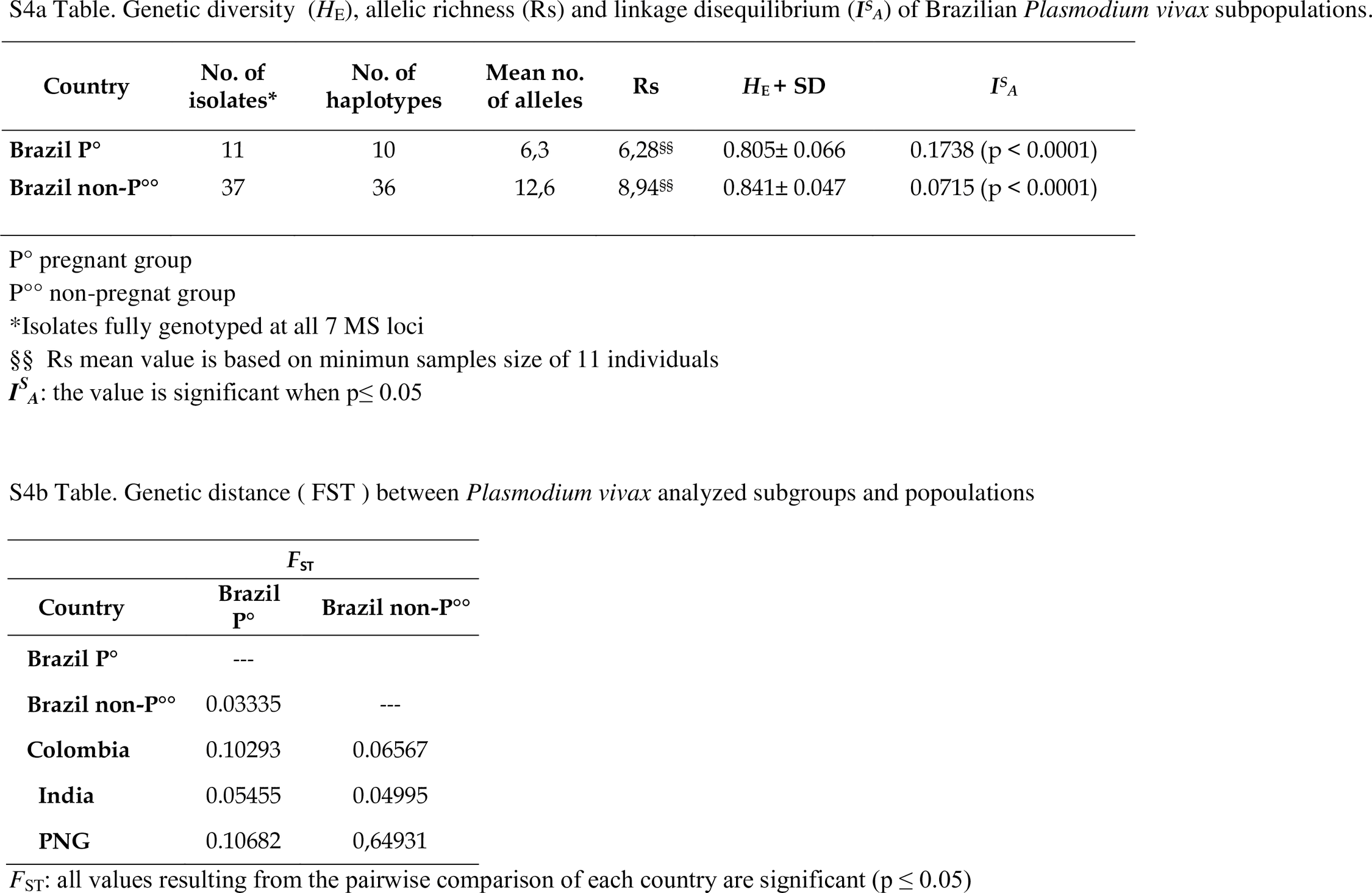

Supplement: S4 Table — (TIF) [file pone.0152447.s005.tif]

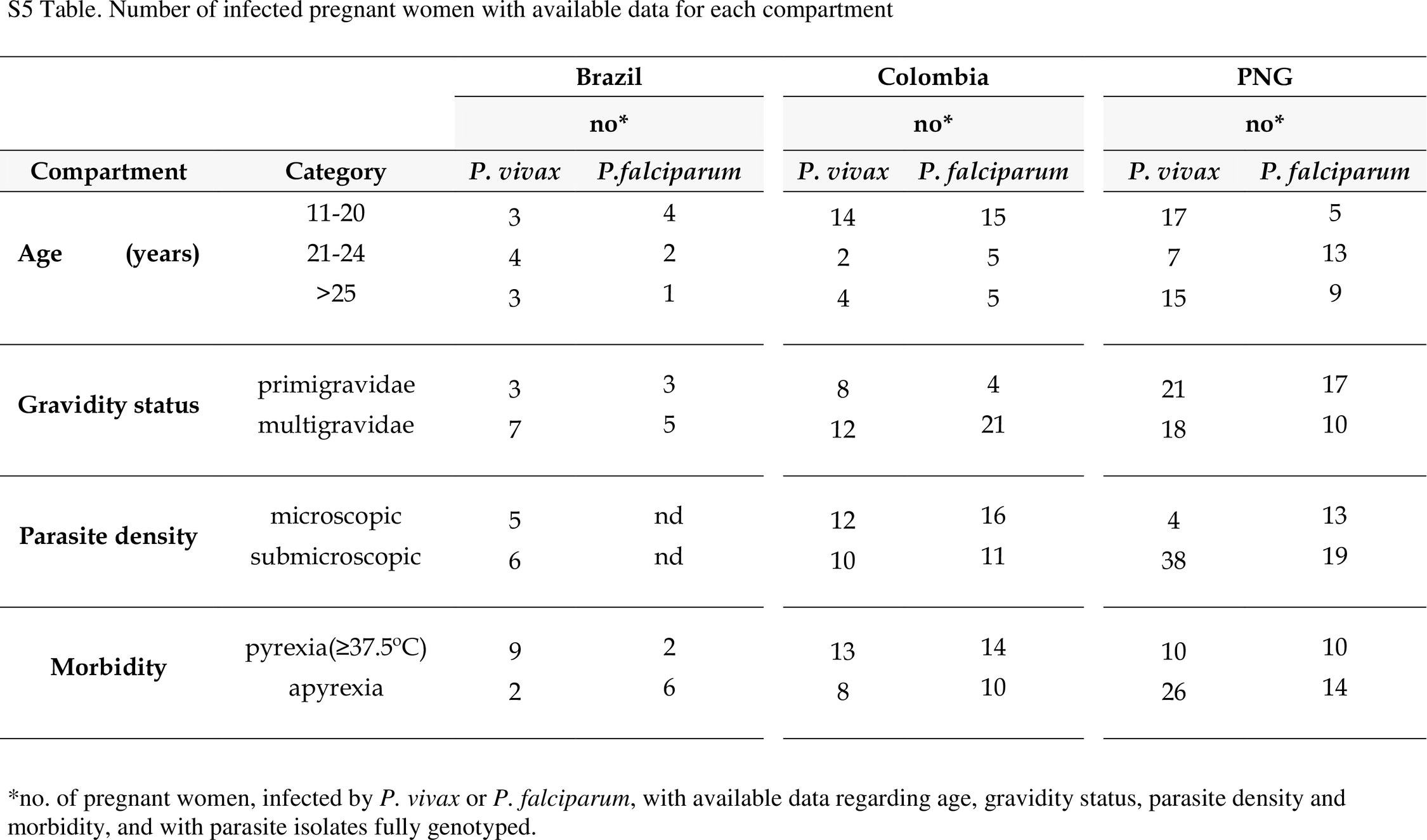

Supplement: S5 Table — (TIF) [file pone.0152447.s006.tif]
